# Ivermectin attenuates *Schistosoma japonicum*-induced liver fibrosis and is associated with downregulation of YAP signaling

**DOI:** 10.3389/fcimb.2025.1678067

**Published:** 2025-10-27

**Authors:** Minhui Xuan, Peiru Zhang, Jiale Guo, Fei Guan, Shengjun Lu, Wenqi Liu

**Affiliations:** Department of Parasitology, School of Basic Medicine, Tongji Medical College, Huazhong University of Science and Technology, Wuhan, China

**Keywords:** ivermectin, *Schistosoma japonicum*, HSCs, YAP, liver fibrosis

## Abstract

Liver fibrosis caused by schistosomiasis is mainly driven by hepatic stellate cell (HSC) activation and excessive extracellular matrix deposition. Yes-associated protein (YAP), a central effector of the Hippo pathway, plays a critical role in HSC activation and liver fibrogenesis. This study investigated the antifibrotic effects of ivermectin, an antiparasitic reported to inhibit YAP, in *Schistosoma japonicum*-infected BALB/c mice. Mice were treated intraperitoneally with ivermectin (2 mg/mL) every two days for four weeks, starting four weeks post-infection. Primary HSCs and Kupffer cells were isolated, and LX-2 cells stimulated with soluble egg antigen (SEA) were treated with ivermectin or cocultured with Kupffer cell supernatants. Histological analysis revealed that ivermectin markedly reduced granuloma formation and collagen deposition. Ivermectin suppressed HSC activation markers, fibrosis-related gene expression, and YAP levels, promoting cytoplasmic retention of phosphorylated YAP. It also reduced arginase-1 and profibrotic markers in Kupffer cells and decreased profibrotic factor secretion in coculture assays. These results demonstrate that ivermectin attenuates *S. japonicum*-induced liver fibrosis and modulates YAP signaling as well as profibrotic activity, highlighting its therapeutic potential in *S. japonicum* infection.

## Introduction

Schistosomiasis is a parasitic disease that significantly threatens human health and economic development, particularly in tropical and subtropical regions. More than 250 million people are infected worldwide, with approximately 80% of cases occurring in Africa, while nearly 800 million individuals remain at risk of infection (Liu et al., 2022; von Bülow et al., 2024). Schistosomiasis is caused by the deposition of eggs in tissues, including the liver and small mesenteric veins, after being transported through the bloodstream. The soluble egg antigen (SEA) released from the eggs primarily triggers Th2-type immune responses, forming egg granulomas (Acharya et al., 2021). In the later stages, these granulomas undergo atrophy as the miracidia within the eggs die and are eventually replaced by fibrosis (McManus et al., 2020).

Hepatic stellate cells (HSCs) are specialized liver cells that play a crucial role in liver regeneration and tissue repair (Ding et al., 2021). Under normal conditions, quiescent HSCs mainly serve as storage sites for retinoids (vitamin A derivatives). However, upon hepatic injury or inflammation, HSCs transdifferentiate into activated myofibroblast-like cells, characterized by increased extracellular matrix (ECM) production, including fibrillar collagen (Gupta et al., 2019). This activation is driven by complex signaling pathways involving various cytokines, such as transforming growth factor-β (TGF-β), platelet-derived growth factor (PDGF), tissue inhibitors of metalloproteinases (TIMPs), and matrix metalloproteinases (MMPs) (Ezhilarasan, 2021). Activated HSCs are typically localized at the periphery of hepatic granulomas, where they exhibit elevated expression of Desmin, α-smooth muscle actin (α-SMA), and glial fibrillary acidic protein (GFAP) (Kisseleva and Brenner, 2021).

Macrophages are categorized into two major subsets based on their effector functions: classically activated M1 and alternatively activated M2. In murine models, M2 macrophages are characterized by the expression of arginase-1 (Arg-1), Ym-1, and FIZZ1/RELMα (Gordon and Martinez, 2010; Hesse et al., 2001). During schistosome infection, M2-polarized macrophages dominate hepatic granulomas (Sandler et al., 2003), secreting TGF-β, which promotes collagen deposition and fibrogenesis (Chuah et al., 2014).

Yes-associated protein (YAP), a critical downstream effector of the Hippo signaling pathway, is widely recognized as an oncogene (Zanconato et al., 2016). Upon activation of the Hippo pathway, YAP is phosphorylated, leading to its cytoplasmic retention and functional inactivation (She et al., 2023). Conversely, when the Hippo pathway is inactivated, YAP is not phosphorylated by LATS1/2, allowing its nuclear translocation. Once in the nucleus, YAP interacts with the TEA domain (TEAD) transcription factors to promote the activation of pro-fibrotic target genes, such as connective tissue growth factor (CTGF) and cardiac anchoring protein repeat domain 1 (ANKRD1) (Yimlamai et al., 2014). YAP activation has been implicated in fibrosis pathogenesis in multiple organs, including the liver (Li et al., 2018; Mannaerts et al., 2015), lung (Lange et al., 2015), kidney (Zhang et al., 2022), and skin (Chitturi et al., 2023). Recent studies have shown that targeting YAP activation through dopamine receptor D1 (Haak et al., 2019) and acid ceramidase (Alsamman et al., 2020) can suppress YAP expression and reduce fibrotic progression, positioning YAP as a potential therapeutic target for fibrotic diseases.

Ivermectin, a dihydro derivative of abamectin, was introduced as an anthelminthic in 1981 and consists of two components: 22,23-dihydro avermectin-B1a (80%) and 22,23-dihydro avermectin-B1b (20%) (Campbell et al., 1983). In addition to its established anti-parasitic effects, ivermectin has shown antiviral (Jans and Wagstaff, 2020), antitumor (Lee et al., 2022), and scabicidal properties (Meyersburg et al., 2023). Accumulating evidence suggests that ivermectin can inhibit YAP activation both *in vitro* and *in vivo* (Nishio et al., 2016). In our study, we developed a murine model of *Schistosoma japonicum* infection to evaluate the potential of ivermectin in mitigating liver fibrosis and suppressing the expression of pro-fibrotic factors. We also investigated the effects of ivermectin on HSC activation and Kupffer cell polarization in response to infection.

## Materials and methods

### *S. japonicum* infection and ivermectin treatment in mice

All animal experiments were conducted according to the guidelines of the Chinese Council on Animal Care and were approved by the Institutional Animal Care and Use Committee (IACUC) of Huazhong University of Science and Technology (Approval No. 2568). Eight-week-old female BALB/c mice were housed under specific pathogen-free (SPF) conditions. To establish the *S. japonicum* infection model, mice were percutaneously exposed to *S. japonicum* cercariae (16 ± 2 per mouse) by immersing the shaved abdominal skin in cercaria-infested water for 30 minutes. Four weeks post-infection, mice were randomly divided into two groups. The treatment group received intraperitoneal injections of ivermectin (2 mg/kg, dissolved in 5% DMSO; MERIAL, France) every 48 h until week 8 post-infection, while the control group received an equivalent volume of 5% DMSO (Sigma, USA) on the same schedule. At week 8 post-infection, all mice were anesthetized using pentobarbital sodium (50 mg/kg, intraperitoneal injection) and subsequently euthanized by cervical dislocation under deep anesthesia. Liver tissues, blood samples, primary HSCs, and Kupffer cells were collected for further analysis.

### Measurement of serum alanine aminotransferase activity

Blood samples were collected from the mice via orbital sinus puncture and allowed to clot spontaneously at room temperature for 2 h. The samples were then centrifuged at 3,500 × g for 10 minutes at 4°C to obtain the serum supernatants. For ALT activity measurement, 20 μl of serum was incubated with a reaction mixture containing α-ketoglutarate and L-alanine at 37°C for 30 minutes to facilitate transamination. Following incubation, 20 μl of 2,4-dinitrophenylhydrazine (2, 4-DNPH) solution was added to each reaction well, and the mixture was further incubated at 37°C for 20 minutes. The reaction was terminated by adding 200 μl of 0.4 mol/L sodium hydroxide and incubating at room temperature for 5 minutes. The OD at 510 nm was measured using a microplate reader, and ALT activity was determined based on a standard calibration curve.

### Liver egg counting

For quantification of *S. japonicum* eggs, approximately 0.1 g of liver tissue was collected from a consistent region of the left hepatic lobe in each mouse. The tissue was homogenized in a 5% KOH solution, and the final volume was adjusted to 20 ml. The homogenate was incubated at 37°C for 30 minutes with continuous shaking at 50 × g to facilitate digestion. After digestion, 200 μl of the processed solution was transferred onto a microscope slide, and egg numbers were counted under a light microscope. The mean egg count was determined from three independent slides. The egg burden per gram (EPG) of liver tissue was calculated using the following formula, EPG =(average egg count×100)/0.1.

### Isolation of primary HSCs and Kupffer cells

Intact livers were excised from mice and dissected into small pieces in PBS. For infected mice, liver tissue was digested with 0.04% Collagenase IV (Sigma, USA) and 0.04% Pronase E (Sigma, USA), while liver tissue from uninfected mice was digested with 0.02% Collagenase IV and 0.02% Pronase E. After 40 minutes of enzymatic digestion, 40 U/ml DNase I (Sigma, USA) was added to the mixture. Following an additional 5 minutes of incubation, the digested tissue was passed through a 40 μm cell strainer and centrifuged at 45 × g for 5 minutes at 4°C. The resulting supernatant, containing non-parenchymal cells (NPCs), was further centrifuged at 500 × g for 5 minutes at 4°C. The obtained NPC pellet was resuspended in PBS and subjected to a two-step Percoll (Solarbio, China) gradient (25%/50%) centrifugation at 1300 × g for 15 minutes at 4°C. Kupffer cells in the intermediate layer were collected. The remaining NPC fraction, after removal of Kupffer cells, was layered onto a 40% Percoll solution and centrifuged at 700 × g for 8 minutes. The intermediate layer was carefully collected into a 15 ml centrifuge tube. To this, 1 ml of 100% Percoll and 4 ml of PBS were added. The mixture was centrifuged again at 700 × g for 6 minutes, and the resulting pellet was collected as primary HSCs. All isolated cells were cultured in an incubator at 37°C with 5% CO2.

### Egg isolation and preparation of SEA

Livers from infected mice were dissected and minced into small pieces. The tissue was homogenized in PBS using a mechanical stirrer. The homogenate was then sequentially filtered through 150 μm and 40 μm mesh sieves to isolate the egg from tissue debris. The residue retained on the 40 μm sieve was collected, resuspended in PBS, and centrifuged at 500 × g for 10 minutes. The supernatant was discarded, and the pellet was resuspended in PBS. This washing procedure was repeated twice to ensure thorough purification. Egg purity was assessed microscopically, and only preparations containing more than 95% intact eggs were used for further processing.

The purified eggs were resuspended in PBS and subjected to five freeze-thaw cycles (from -80°C to 4°C) to disrupt cellular structures. The suspension was then sonicated at 400 W until complete eggshell rupture was achieved. The lysate was subsequently centrifuged at 10,000 × g for 30 minutes at 4°C to separate the soluble fraction. The supernatant, containing SEA, was filtered through a 0.22 μm sterile filter to remove any remaining particulates. The protein concentration of SEA was quantified using a BCA protein assay kit (Beyotime, China). The resulting SEA preparation was stored at -80°C for subsequent use.

### Cell culture and SEA or ivermectin treatment

The LX-2 cell line was stored and maintained in our laboratory. Cells were cultured in a complete medium consisting of 89% DMEM (Gibco, USA), 10% fetal bovine serum (Gibco, USA), and 1% streptomycin/penicillin (Beyotime, China). All cells were maintained at 37°C in a humidified incubator with 5% CO2. Primary HSCs were isolated from normal mouse livers using a density gradient centrifugation protocol, as described above. After isolation, both primary HSCs and LX-2 cells were seeded into six-well plates at a density of 2 × 10^5^ cells per well. After 24 h of adhesion, the cells were pre-treated with SEA at a concentration of 5 μg/mL for 48 h to induce activation, followed by treatment with ivermectin (10 μM) for an additional 24 h.

### LX-2 treatment with Kupffer’s supernatant

Primary Kupffer cells were isolated from the livers of *S. japonicum*-infected mice and ivermectin-treated mice. After 48 h of *in vitro* incubation, the culture supernatants were collected. LX-2 cells were serum-deprived in DMEM for 24 h to synchronize cell growth. They were then treated with supernatants derived from either infected or ivermectin-treated Kupffer cells for 72 h. Total RNA was extracted from LX-2 cells, followed by reverse transcription for further analysis.

### Real-time PCR

Total RNA was extracted from tissues or purified cells using TRIzol reagent (Yeasen, China) and reverse-transcribed into complementary DNA (cDNA) using a reverse transcription kit (TOYOBO, Japan), following the manufacturer’s instructions. qPCR reactions were prepared in a total volume of 20 μl, consisting of 10 μl SYBR^®^ Premix Ex Taq™ (Takara, Japan), 0.2 μl reverse transcription product, 0.4 μl forward primer, 0.4 μl reverse primer, and 9 μl distilled water (Yeasen, China). The reaction mixtures were loaded onto a MyiQ™2 Real-time PCR System (Bio-Rad, USA). Gene expression levels were normalized to the housekeeping gene glyceraldehyde-3-phosphate dehydrogenase (GAPDH) and calculated using the 2^−ΔΔCT^ method. Primer sequences are listed in **Table 1**.

**Table 1:** Primer sequences of mouse genes used in RT-PCR.

Gene	Forward primer (5’-3’)	Reverse primer (5’-3’)
*GAPDH*	GTGTTTCCTCGTCCCGTAG	ATGGCAACAATCTCCACTTT
*TGF-β*	CCCACTGATACGCCTGAT	GGGCTGATCCCGTTGAT
*PPAR-γ*	CTCCAAGAATACCAAAGTGCGA	GCCTGATGCTTTATCCCCACA
*α-SMA*	CCCAGACATCAGGGAGTAATGG	TCTATCGGATACTTCAGCGTCA
*ANKRD1*	TGCGATGAGTATAAACGGACG	GTGGATTCAAGCATATCTCGGAA
*YAP1*	ACCCTCGTTTTGCCATGAAC	TTGTTTCAACCGCAGTCTCTC
*COL1A1*	GGCGGTTCAGGTCCAAT	TCGGTGTCCCTTCATTCC
*CTGF*	TGTGAAGACATACAGGGCTAAG	ACAGTTGTAATGGCAGGCAC
*Arg1*	CTCCAAGCCAAAGTCCTTAGAG	GGAGCTGTCATTAGGGACATCA
*Desmin*	AGACCATCGCGGCTAAGAAC	ATCAGGGAATCGTTAGTGCCC

### Western blot

Proteins were extracted from cells or tissues using RIPA lysis buffer (Beyotime, China), supplemented with phenylmethylsulfonyl fluoride (PMSF, Sigma, USA) to inhibit protease activity. The extracted proteins were quantified, denatured, and separated by sodium dodecyl sulfate-polyacrylamide gel electrophoresis (SDS-PAGE). The separated proteins were then transferred onto polyvinylidene difluoride (PVDF) membranes (Sigma, USA). Following the transfer, the membranes were blocked with 5% bovine serum albumin (BSA) in TBST for 1 hour at room temperature to minimize nonspecific binding. Subsequently, the membranes were incubated overnight at 4°C with the following primary antibodies: anti-YAP (1:5000, Proteintech, USA), anti-P-YAP-Ser127 (1:2000, Cell Signaling Technology, USA), anti-Desmin (1:5000, Proteintech, USA), anti-α-SMA (1:2500, Proteintech, USA), and anti-GAPDH (1:5000, Proteintech, USA), which served as a loading control. After three washes with TBST (10 minutes per wash), the membranes were incubated with HRP-conjugated secondary antibodies (diluted 1:2000 in 5% BSA) for 2 h at room temperature. After additional TBST washes, protein bands were detected using an enhanced chemiluminescence (ECL) reagent (Beyotime, China) and visualized with a Bio-Rad ChemiDoc™ imaging system.

### Liver histopathology

Liver tissues were fixed in 4% paraformaldehyde at room temperature for 24 h, followed by dehydration, paraffin embedding, and sectioning at a thickness of 5 μm. The sections were stained with hematoxylin and eosin (H&E) to assess overall tissue morphology and histopathological changes. To evaluate collagen deposition and fibrosis, sections were stained with Masson’s trichrome. Histological images were acquired using the Mshot Image Analysis System (Guangzhou Micro-shot Technology Co., Ltd, Guangzhou, China) and analyzed for further evaluation.

### Immunofluorescence

Primary HSCs, LX-2 cells, and liver tissue sections were fixed with 4% paraformaldehyde for 15 minutes at room temperature, followed by permeabilization with 0.1% Triton X-100 for 10 minutes. After blocking with goat serum for 30 minutes, the samples were incubated overnight at 4°C with primary antibodies against COL1A1 (1:200, Proteintech, USA), TGF-β (1:200, Proteintech, USA), YAP (1:200, Proteintech, USA), and α-SMA (1:200, Proteintech, USA). The next day, sections were incubated with a fluorescently labeled secondary antibody (1:100, Proteintech, USA) at room temperature for 1 hour. Nuclei were counterstained with DAPI for 5 minutes, and samples were mounted with the antifade medium. Fluorescent images were acquired using a fluorescence microscope.

### Statistical analysis

All data are presented as the mean ± SEM. Statistical analyses were performed using SPSS 24.0 software. Comparisons between two groups were conducted using an independent-samples t-test, while comparisons among three or more groups were performed using one-way ANOVA. *P* < 0.05 was considered statistically significant.

## Results

### Ivermectin reduces liver fibrosis caused by *S. japonicum* infection

**Figure 1:**
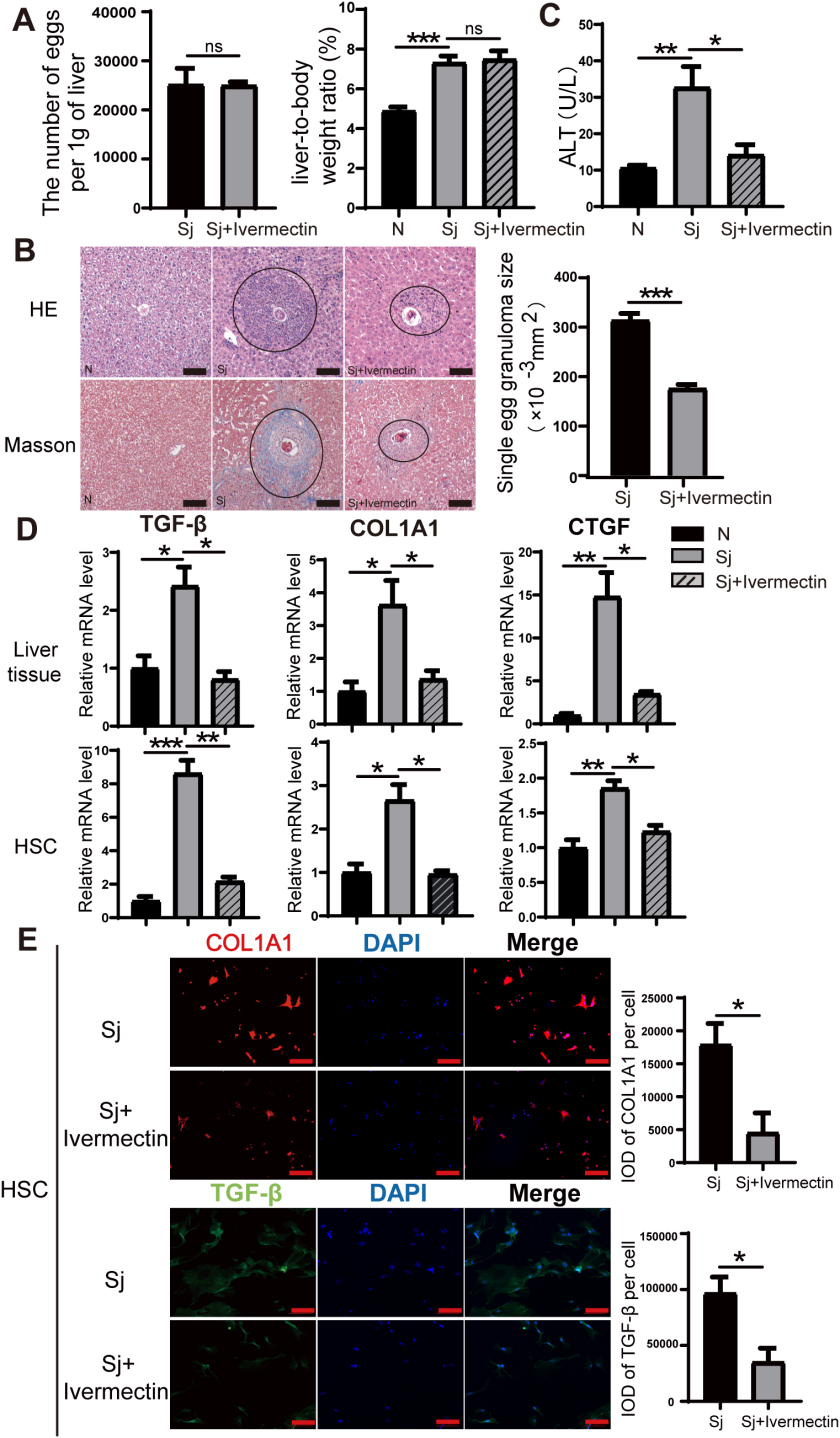
Ivermectin alleviates liver fibrosis induced by *S. japonicum* infection. **(A)** Quantification of egg burden (eggs per gram of liver) and liver-to-body weight ratio in infected and treated mice. **(B)** Representative HE and Masson staining images of hepatic granulomas in mice 8 weeks after *S. japonicum* infection. The average size of individual granulomas was measured. Scale bars: 100 μm. **(C)** Serum ALT levels in different experimental groups. **(D)** Relative mRNA expression levels of fibrosis markers (TGF-β, COL1A1, and CTGF) in liver tissue (top) and primary HSCs (bottom). **(E)** Immunofluorescence staining for COL1A1 and TGF-β in primary HSCs. Scale bars: 100 μm. All data were presented as mean ± SEM. Statistical significance was determined using Student’s t-test for two-group comparisons (*P < 0.05, **P < 0.01, ***P < 0.001, ns, no statistical significance).

Following *S. japonicum* infection and subsequent ivermectin treatment, no significant differences were observed in hepatic egg burden (eggs per gram of liver) or the liver-to-body weight ratio between the infected and ivermectin-treated groups (**Figure 1A**), indicating that ivermectin does not affect *S. japonicum* oviposition. Histopathological evaluation using HE and Masson’s trichrome staining revealed that ivermectin treatment markedly reduced inflammatory cell infiltration surrounding schistosome eggs and significantly decreased collagen fiber deposition, indicating an improvement in liver fibrosis (**Figure 1B**). Serum ALT, a key biomarker of acute liver injury, was also measured and showed reduced levels following ivermectin treatment, suggesting potential hepatoprotective effects (**Figure 1C**). To further investigate the anti-fibrotic effects of ivermectin, we examined the mRNA expression of key profibrotic markers (TGF-β, COL1A1, and CTGF) in liver tissue via RT-PCR. These genes were significantly upregulated following *S. japonicum* infection but were notably downregulated after ivermectin treatment (**Figure 1D**). To validate these findings at the cellular level, primary HSCs were isolated and purified from mice, and the expression of TGF-β, COL1A1, and CTGF was assessed. Consistently, the expression in HSCs mirrored the trends observed in liver tissues (**Figure 1D**). In addition, immunofluorescence staining demonstrated that ivermectin significantly reduced COL1A1 and TGF-β protein expression in primary HSCs after infection, further corroborating its antifibrotic effects (**Figure 1E**). Collectively, these findings indicate that ivermectin treatment effectively attenuates liver fibrosis induced by *S. japonicum* infection.

### YAP expression is upregulated in mice infected with *S. japonicum*

**Figure 2:**
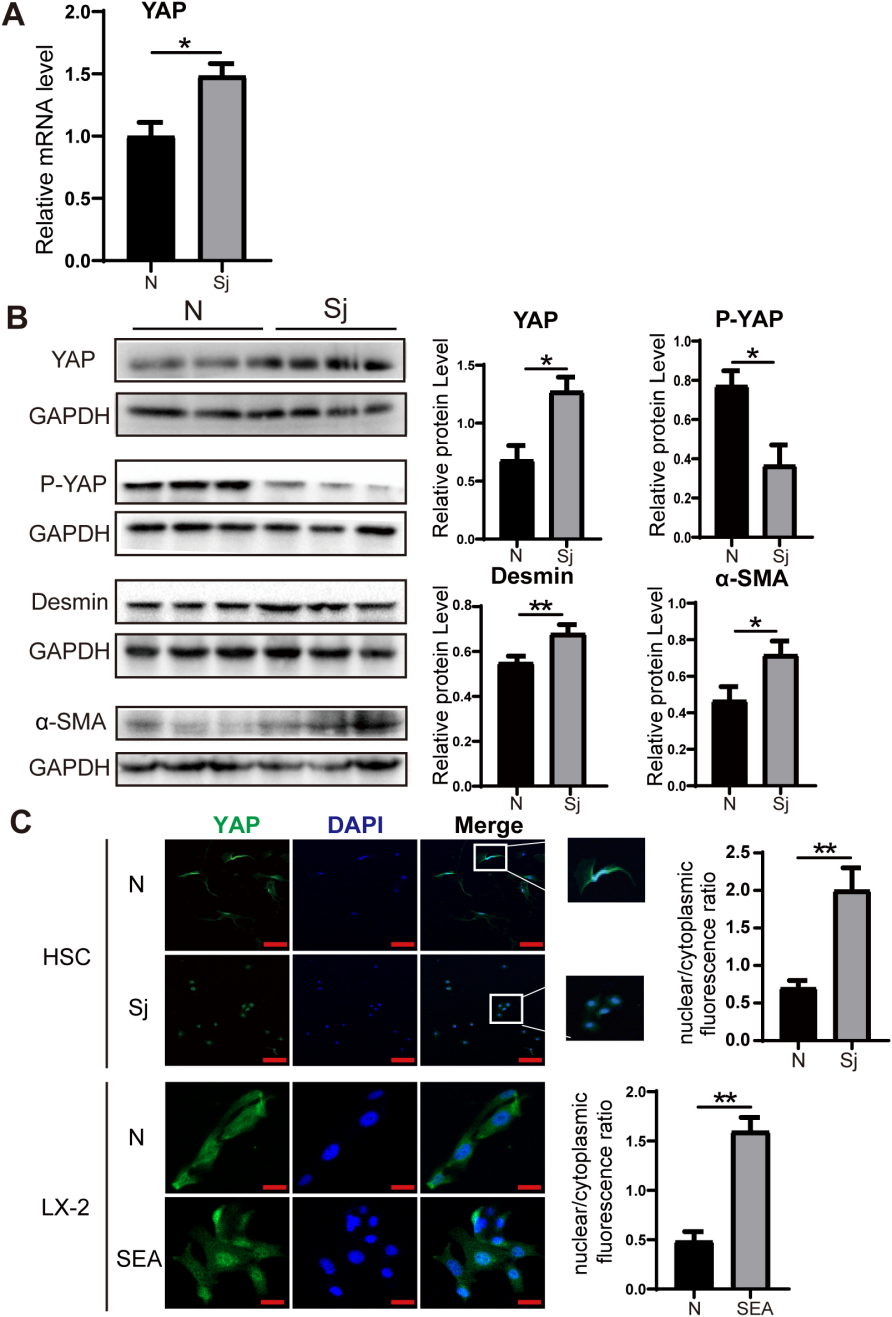
Increased expression of YAP in *S. japonicum*-infected mice. **(A)** RT-PCR analysis showing increased YAP mRNA expression level in liver tissue. **(B)** Western blot analysis of YAP, P-YAP, Desmin, and α-SMA in liver tissue. **(C)** Immunofluorescence staining showing YAP expression and nuclear-to-cytoplasmic ratio in primary HSCs (top) and LX-2 cells (bottom). Scale bars: 100 μm (up), 20μm (down). All data were expressed as mean ± SEM. Statistical significance was determined using Student’s t-test for two-group comparisons (*P < 0.05, **P < 0.01, ns, no statistical significance).

After 8 weeks of infection, liver tissues were collected, and primary HSCs were isolated. RT-PCR analysis revealed a significant upregulation of YAP mRNA expression in liver tissues from infected mice compared to the uninfected group (**Figure 2A**). Western blot analysis further demonstrated increased protein levels of YAP and HSC activation markers (Desmin and α-SMA) following infection, while phosphorylated YAP (p-YAP) expression was reduced (**Figure 2B**). Immunofluorescence analysis of primary HSCs showed that YAP was predominantly localized in the nucleus in infected mice. Moreover, the nuclear-to-cytoplasmic ratio of YAP was significantly higher in infected mice than in uninfected mice. A similar trend was observed in LX-2 cells treated with SEA (**Figure 2C**). These findings indicate that YAP is highly expressed and preferentially localized to the nucleus, which correlates with significant HSC activation in mice following *S. japonicum* infection.

### Ivermectin inhibits HSC activation in *S. japonicum*-infected mice and reduces YAP expression

**Figure 3:**
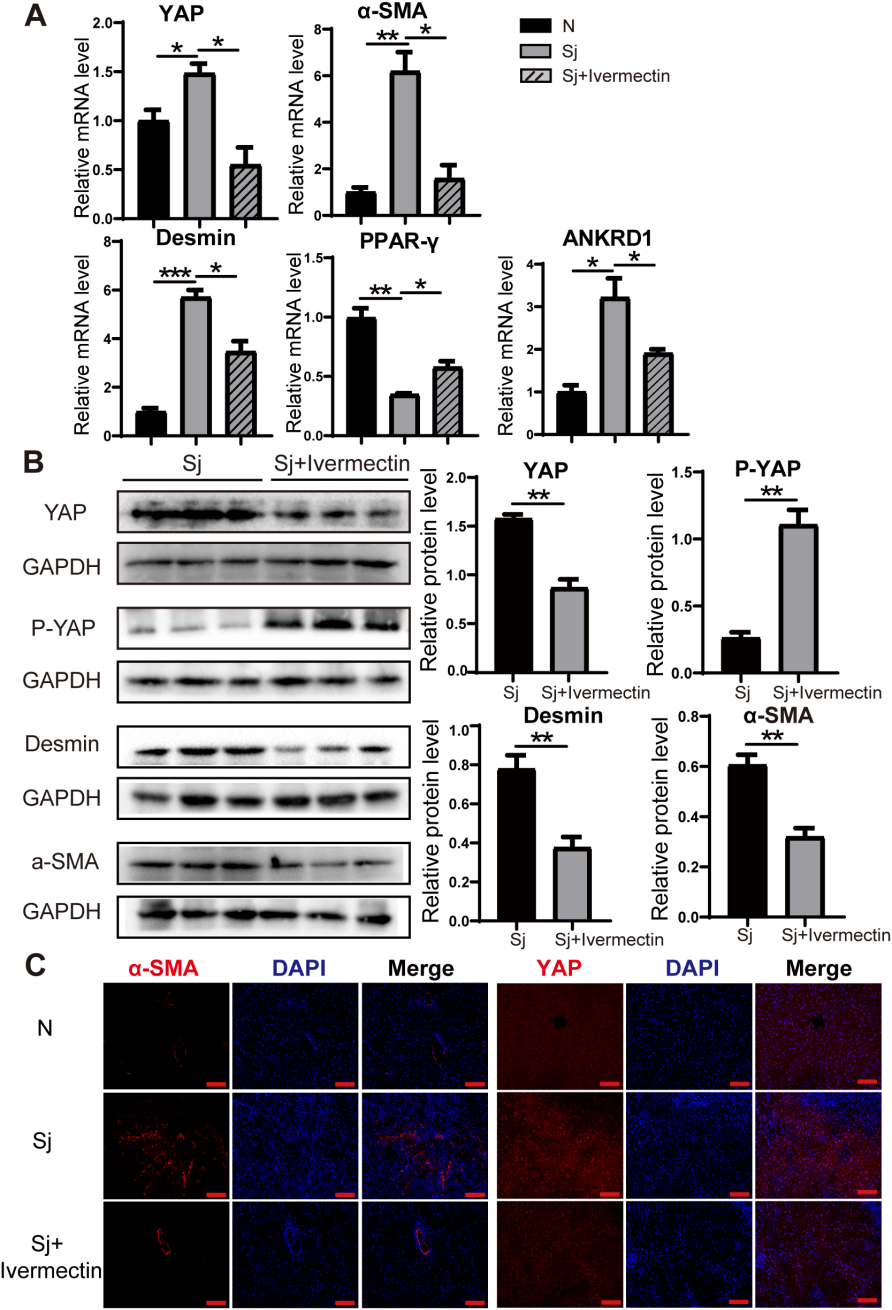
Ivermectin reduced YAP levels and inhibited HSC activation in *S. japonicum*-infected mice. **(A)** RT-PCR analysis of YAP, α-SMA, Desmin, PPAR-γ, and ANKRD1 mRNA levels in liver tissue. **(B)** Western blot analysis of YAP, P-YAP, Desmin, and α-SMA protein levels. **(C)** Immunofluorescence staining of α-SMA and YAP in liver tissue. Scale bars: 100 μm. All data were expressed as mean ± SEM. Statistical significance was determined using Student’s t-test for two-group comparisons (*P < 0.05, **P < 0.01, ***P < 0.001, ns, no statistical significance).

To investigate whether ivermectin inhibits HSC activation and affects YAP expression in infected mice, we isolated liver tissues and analyzed the mRNA levels of YAP, the quiescent HSC marker (PPAR-γ), the activation markers (α-SMA, Desmin), and the YAP downstream target gene (ANKRD1) via RT-PCR. Notably, in ivermectin-treated infected mice, YAP, α-SMA, Desmin, and ANKRD1 mRNA levels were significantly reduced, while PPAR-γ expression was upregulated (**Figure 3A**). Similarly, western blot analysis demonstrated that ivermectin decreased YAP, α-SMA, and Desmin protein levels while increasing P-YAP expression (**Figure 3B**). Immunofluorescence staining of liver tissues further confirmed that α-SMA and YAP protein levels were elevated following infection but downregulated after ivermectin treatment (**Figure 3C**).

**Figure 4:**
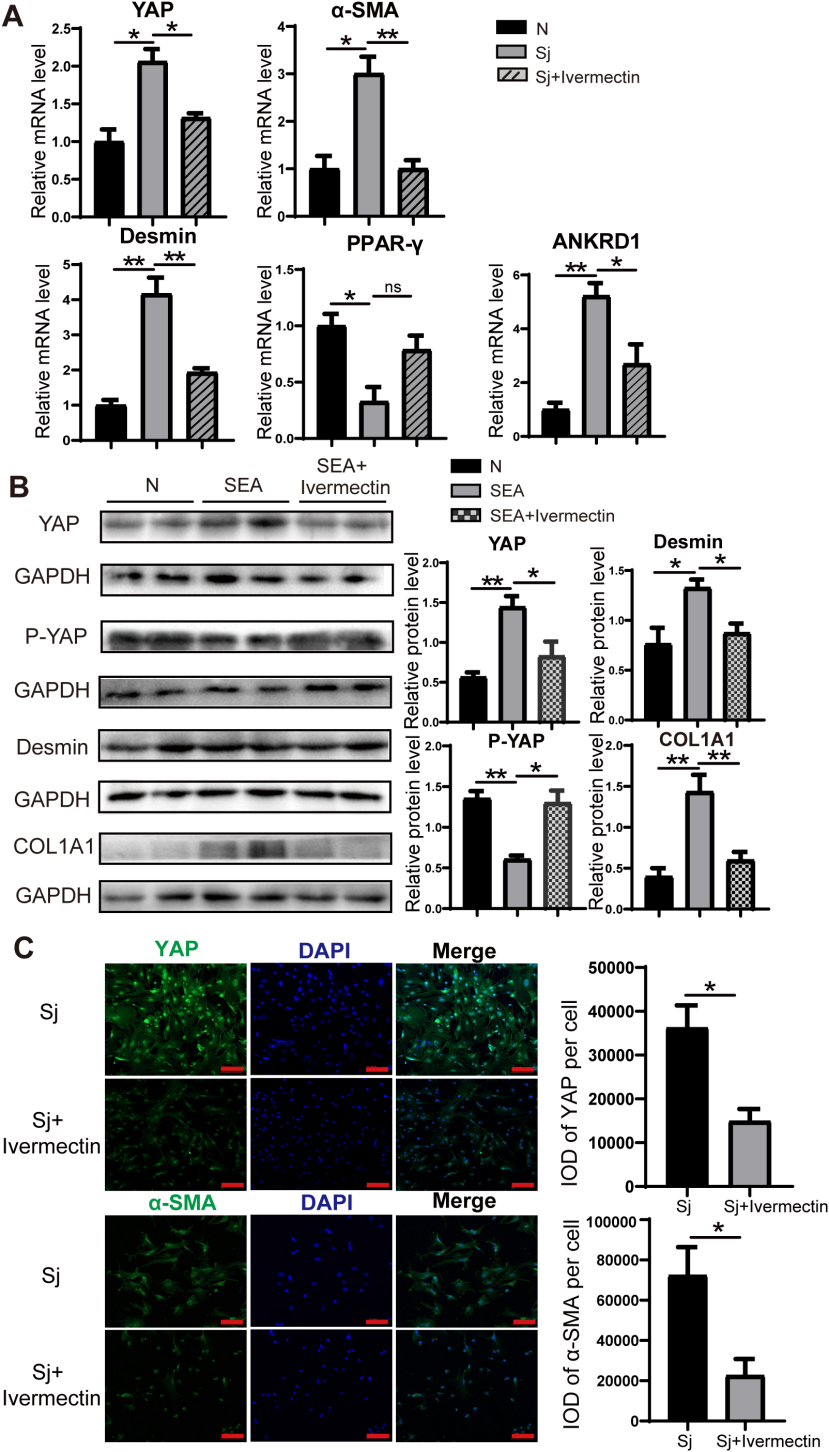
Ivermectin reduced YAP levels and inhibited the HSC activation in primary HSCs. **(A)** RT-PCR analysis of YAP, α-SMA, Desmin, PPAR-γ, and ANKRD1 mRNA levels in primary HSCs. **(B)** Western blot analysis of YAP, P-YAP, Desmin, and COL1A1 protein levels in primary HSCs. **(C)** Immunofluorescence staining of YAP (top) and α-SMA (bottom) in primary HSCs. Scale bars: 100 μm. All data were expressed as mean ± SEM. Statistical significance was determined using Student’s t-test for two-group comparisons (*P < 0.05, **P < 0.01, ns, no statistical significance).

To further evaluate the effects of ivermectin on HSCs, we first isolated primary HSCs from *S. japonicum*-infected mice treated with or without ivermectin. RT-PCR analysis showed that ivermectin treatment markedly reduced the expression of YAP, α-SMA, Desmin, and ANKRD1, although PPAR-γ levels were not significantly upregulated (**Figure 4A**). In addition, immunofluorescence analysis confirmed that YAP and α-SMA protein expression were decreased in HSCs from ivermectin-treated mice (**Figure 4C**). To investigate the direct effects of ivermectin on HSCs in a controlled setting, we isolated primary HSCs from normal mice and performed *in vitro* stimulation. The cells were stimulated by SEA treatment and then exposed to ivermectin. Western blot results showed that SEA increased YAP, Desmin, and COL1A1 levels while decreasing P-YAP expression, and these effects were reversed by ivermectin (**Figure 4B**). Collectively, these results from liver tissues and primary HSCs indicate that ivermectin reduces YAP expression and inhibits HSC activation in *S. japonicum*-infected mice.

### Ivermectin suppresses SEA-induced activation of LX-2 cells and downregulates YAP expression

**Figure 5:**
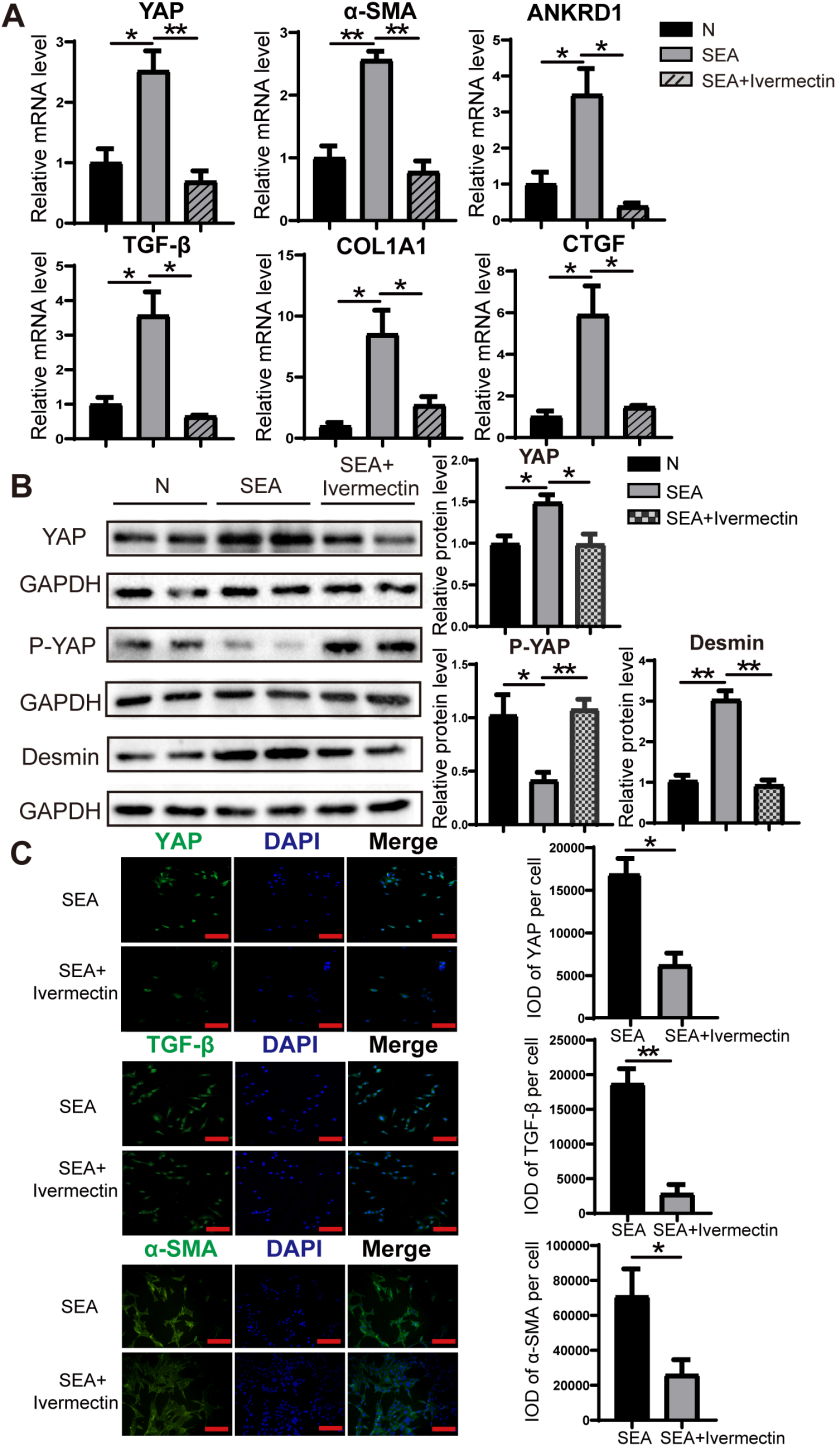
Ivermectin reduced YAP levels and inhibited LX-2 cell activation following SEA treatment. **(A)** RT-PCR analysis of YAP, α-SMA, ANKRD1, TGF-β, COL1A1, and CTGF mRNA levels in LX-2 cells. **(B)** Western blot analysis of YAP, P-YAP, and Desmin protein levels in LX-2 cells. **(C)** Immunofluorescence staining of YAP, TGF-β, and α-SMA in LX-2 cells. All data were expressed as mean ± SEM. Statistical significance was determined using Student’s t-test for two-group comparisons (*P < 0.05, **P < 0.01, ns, no statistical significance).

To evaluate the effect of ivermectin on SEA-induced activation of LX-2 cells, we cultured LX-2 cells in the presence of SEA with or without ivermectin. RT-PCR analysis revealed that SEA stimulation significantly increased the expression of YAP, α-SMA, ANKRD1, TGF-β, COL1A1, and CTGF, whereas ivermectin treatment effectively mitigated this upregulation (**Figure 5A**). Consistently, western blot analysis demonstrated that ivermectin reduced the protein levels of YAP and Desmin while promoting the expression of P-YAP (**Figure 5B**). Immunofluorescence staining further confirmed that ivermectin treatment effectively diminished the expression of YAP, TGF-β, and α-SMA at the protein level (**Figure 5C**). These findings indicate that ivermectin inhibits SEA-induced LX-2 cell activation, potentially through the downregulation of YAP signaling.

### Ivermectin reduces the expression of representative M2 marker factors in Kupffer cells of *S. japonicum*-infected mice, attenuating their ability to activate HSCs

**Figure 6:**
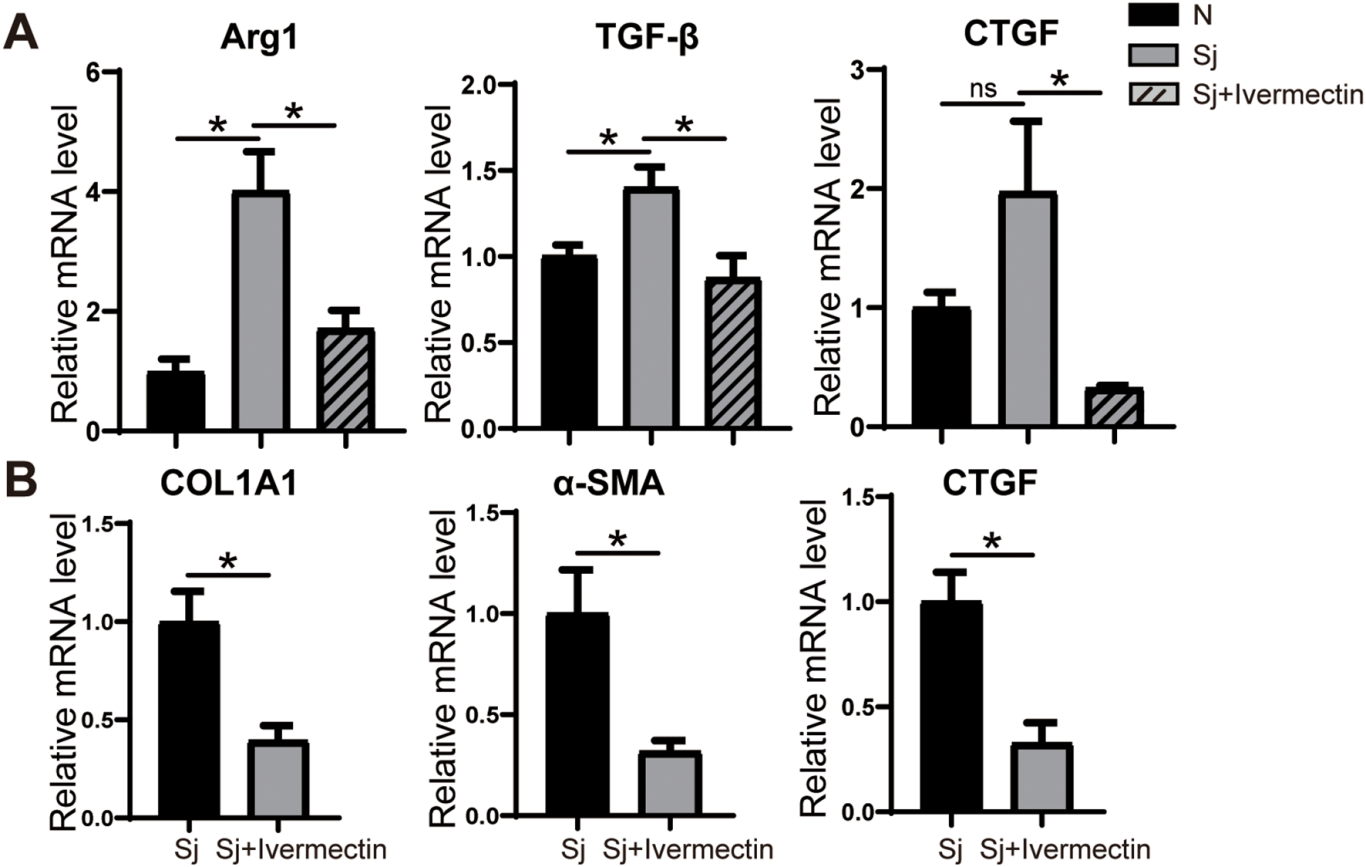
Ivermectin inhibited the differentiation of liver Kupffer cells into M2 macrophages in *S. japonicum*-infected mice. **(A)** RT-PCR analysis of Arg1, TGF-β, and CTGF mRNA levels in primary Kupffer cells. **(B)** RT-PCR analysis of COL1A1, α-SMA, and CTGF mRNA levels in LX-2 cells treated with Kupffer cell supernatant. All data were expressed as mean ± SEM. Statistical significance was determined using Student’s t-test for two-group comparisons (*P < 0.05, ns, no statistical significance).

To investigate whether ivermectin modulates the phenotype of Kupffer cells, primary Kupffer cells were isolated from mice infected with *S. japonicum* and analyzed for gene expression using RT-PCR. The results demonstrated a significant downregulation of Arg1, a key marker of M2 polarization, along with the pro-fibrotic factors TGF-β and CTGF in Kupffer cells from the ivermectin-treated group compared to untreated controls (**Figure 6A**). Furthermore, co-culture of LX-2 cells with supernatants derived from primary Kupffer cells of infected or ivermectin-treated mice revealed a marked reduction in the expression levels of COL1A1, α-SMA, and CTGF in the ivermectin-treated group (**Figure 6B**). These findings suggest that ivermectin inhibits M2 polarization of Kupffer cells and suppresses their secretion of pro-fibrotic factors, thereby limiting their capacity to activate HSCs in the context of schistosome infection.

## Discussion

In a healthy liver, HSCs remain in a quiescent state, serving as the primary reservoir for retinoids and vitamin A-containing metabolites (Kamm and McCommis, 2022). Upon liver injury, quiescent HSCs undergo a phenotypic transition characterized by the downregulation of retinoid storage-related genes, GFAP, and PPAR-γ, along with the upregulation of Desmin, α-SMA, and intracellular microfilaments, ultimately leading to their activation into myofibroblast-like cells (Carson et al., 2018; Müller et al., 2024; You et al., 2023). TGF-β is a critical pro-fibrotic cytokine that drives HSC activation through a SMAD2/3-dependent signaling pathway (Xu et al., 2016). Additionally, CTGF has been shown to enhance COL1A1 expression in activated HSCs via a TGF-β-independent mechanism (Liu et al., 2011). In this study, we demonstrated that HSC activation occurs following *S. japonicum* infection in mice or exposure to SEA in LX-2 cells. This activation was evidenced by the upregulation of Desmin and α-SMA at both mRNA and protein levels, accompanied by increased expression of key pro-fibrotic markers, including TGF-β, CTGF, and COL1A1. These findings underscore the pivotal role of HSC activation in schistosomiasis-induced hepatic fibrosis.

YAP, a key downstream effector of the Hippo signaling pathway, plays a crucial role in organ growth, tissue renewal, regeneration, and cell proliferation (Piccolo et al., 2014). Under normal physiological conditions, activation of the Hippo pathway leads to the phosphorylation of YAP, retaining it in the cytoplasm and preventing its transcriptional activity. However, when Hippo pathway kinases are inactivated, YAP translocates to the nucleus, where it promotes the expression of target genes, such as CTGF and ANKRD1, thereby driving fibrotic responses (Moroishi et al., 2015). Both human and murine myofibroblasts, as well as stellate cells, exhibit significant nuclear accumulation of YAP in fibrotic lungs, hearts, and livers (Du et al., 2023; Garoffolo et al., 2022; Zhu et al., 2020). YAP has been identified as a crucial regulator of HSC activation and plays a central role in the progression of hepatic fibrosis and cirrhosis (Xiang et al., 2020). Notably, in mouse models of liver fibrosis, pharmacological inhibition of YAP effectively suppressed myofibroblast accumulation and fibrosis progression (Du et al., 2018). Our study demonstrated that *S. japonicum* infection in mice and SEA stimulation in LX-2 cells led to increased YAP expression, accompanied by a reduction in the P-YAP levels at the protein level. Furthermore, we observed a significant increase in the nuclear-to-cytoplasmic ratio of YAP, alongside the upregulation of its downstream effectors. These findings indicate that YAP is critical for HSC activation, making YAP an essential target for the treatment of liver fibrosis. However, we acknowledge that while the changes in YAP and P-YAP expression observed in SEA-stimulated primary HSCs suggest YAP pathway activation, the current study does not include functional inhibition experiments (e.g., YAP knockdown or inhibitor use) to directly establish causality. Future studies incorporating targeted modulation of YAP activity will be essential to definitively confirm its mechanistic role in ivermectin-mediated suppression of HSC activation and liver fibrosis.

Notably, our *in vitro* model showed that SEA alone was able to activate primary HSCs from healthy mice, as shown by increased YAP and Desmin expression and reduced P-YAP levels. Although immune cells contribute to HSC activation *in vivo*, previous studies have confirmed that SEA can directly activate HSCs via TLR4 and pro-fibrotic signaling pathways, including P38 and PI3K/AKT, even in the absence of immune cells (Liu et al., 2013; Wen et al., 2017). These findings support the use of SEA-treated HSCs as a valid model for studying fibrogenic mechanisms and drug responses *in vitro*.

Ivermectin is a macrolide compound with broad-spectrum anti-parasitic activity (Laing et al., 2017). It is widely used in the treatment of river blindness, elephantiasis, scabies, and other parasitic infections. However, ivermectin is not an effective agent against schistosomiasis in humans, as it does not kill the adult parasites. Beyond its antiparasitic properties, recent studies have revealed that ivermectin also exhibits antitumor, antiviral, and anti-inflammatory effects (Diao et al., 2019; DiNicolantonio et al., 2020; Yang et al., 2020). Despite these emerging findings, the molecular mechanisms underlying the effects of ivermectin remain poorly understood, and rigorous clinical trials validating these effects are still lacking or provide insufficient data. Therefore, further in-depth investigations are necessary to elucidate the mechanistic pathways of ivermectin in these novel therapeutic contexts and to assess its potential clinical applications.

In this study, we provide the first evidence that ivermectin influences the progression of liver fibrosis in mice infected with *S. japonicum*. Specifically, we assessed liver-to-body weight ratios in both infected and uninfected mice, revealing a significant enlargement of the liver in infected mice. HE and Masson staining demonstrated substantial eosinophilic infiltration and prominent collagen fiber deposition in the livers of infected mice, indicative of significant fibrosis following *S. japonicum* infection. To evaluate the therapeutic potential of ivermectin, we initiated treatment with ivermectin at the end of the fourth week post-infection. At this time, egg antigens begin to penetrate host tissues, triggering progressive fibrosis responses. Notably, ivermectin treatment resulted in a marked reduction in the area of worm egg granulomas in the liver compared to the untreated infected group. Furthermore, fibrosis-associated markers, including TGF-β, CTGF, and COL1A1, were significantly downregulated in both liver tissues and primary HSCs following ivermectin administration. These results suggest that ivermectin mitigates liver fibrosis in schistosome-infected mice, potentially through modulation of the fibrotic signaling cascade.

In recent years, several studies have demonstrated that ivermectin effectively targets YAP. For instance, in *Mob1*-deficient mice, ivermectin was shown to inhibit YAP1 activation and downregulate CTGF expression, resulting in reduced hepatic enlargement and decreased accumulation of oval cells and immature bile duct cells (Nishio et al., 2016). These findings suggest that ivermectin acts as a YAP inhibitor and effectively suppresses elevated YAP expression, which is consistent with our results. In our study, we identified YAP suppression as a primary mechanism by which ivermectin attenuates hepatic fibrosis in schistosome-infected mice. Specifically, ivermectin treatment led to a significant reduction in YAP expression, which subsequently inhibited HSC activation. These results highlight the potential of ivermectin as a therapeutic agent for schistosomiasis-induced liver fibrosis by targeting YAP-mediated fibrotic signaling.

Liver macrophages consist of ontogenically distinct populations, primarily Kupffer cells and monocyte-derived macrophages (Tacke, 2017). Among these, Kupffer cells represent the largest population of resident macrophages in the liver and play a crucial role in maintaining homeostasis in an uninjured liver (Ju and Pohl, 2005). Kupffer cells are a primary source of TGF-β, and their overexpression contributes to fibrosis progression (Bonniaud et al., 2005). In models of CCL_4_-induced chronic liver injury, depletion of Kupffer cells during active injury has been shown to attenuate fibrosis (Sunami et al., 2012). Previous studies indicate that downregulation of YAP expression inhibits macrophage polarization toward the M2 phenotype, as evidenced by a significant reduction in M2 markers (CD23 and Arg1), while M1 markers (CD64 and CCR7) remain unaffected (Huang et al., 2017). Consistent with this, our study found that ivermectin suppressed M2 polarization of Kupffer cells, likely via modulation of YAP signaling, thereby mitigating their pro-fibrotic effects. In addition to Arg-1, other M2 markers such as CD163 and CD206 may provide further insight into macrophage polarization status. Future studies incorporating a broader marker panel would help to better define M2 macrophage subsets in schistosomiasis-related liver fibrosis. In conclusion, these findings suggest that ivermectin may modulate Kupffer cell-mediated activation of HSCs through cytokine secretion.

## Conclusions

Using a mouse model of *S. japonicum* infection and SEA-treated LX-2 cells, this study provides compelling evidence that ivermectin attenuates the liver fibrosis induced by *S. japonicum* infection. The primary mechanism underlying its anti-fibrotic effects appears to involve the inhibition of the YAP pathway, which subsequently reduces HSC activation and suppresses the polarization of Kupffer cells toward the M2 phenotype. These findings suggest that ivermectin may serve as a promising therapeutic agent for mitigating liver fibrosis associated with schistosomiasis and other fibrotic liver diseases. However, further research is required to assess its therapeutic efficacy and safety in human patients.

## Data Availability

The original contributions presented in the study are included in the article/supplementary material. Further inquiries can be directed to the corresponding author.

## References

[B1] Acharya S. Da'dara A. A. Skelly P. J. (2021). Schistosome immunomodulators. PloS Pathog. 17, e1010064. doi: 10.1371/journal.ppat.1010064, PMID: 34969052 PMC8718004

[B2] Alsamman S. Christenson S. A. Yu A. Ayad N. M. E. Mooring M. S. Segal J. M.. (2020). Targeting acid ceramidase inhibits YAP/TAZ signaling to reduce fibrosis in mice. Sci. Transl. Med. 12, eaay8798. doi: 10.1126/scitranslmed.aay8798, PMID: 32817366 PMC7976849

[B3] Bonniaud P. Margetts P. J. Ask K. Flanders K. Gauldie J. Kolb M. (2005). TGF-beta and Smad3 signaling link inflammation to chronic fibrogenesis. J. Immunol. 175, 5390–5395. doi: 10.4049/jimmunol.175.8.5390, PMID: 16210645

[B4] Campbell W. C. Fisher M. H. Stapley E. O. Albers-Schönberg G. Jacob T. A. (1983). Ivermectin: a potent new antiparasitic agent. Science 221, 823–828. doi: 10.1126/science.6308762, PMID: 6308762

[B5] Carson J. P. Ramm G. A. Robinson M. W. McManus D. P. Gobert G. N. (2018). Schistosome-induced fibrotic disease: the role of hepatic stellate cells. Trends Parasitol. 34, 524–540. doi: 10.1016/j.pt.2018.02.005, PMID: 29526403

[B6] Chitturi P. Xu S. Ahmed Abdi B. Nguyen J. Carter D. E. Sinha S.. (2023). Tripterygium wilfordii derivative celastrol, a YAP inhibitor, has antifibrotic effects in systemic sclerosis. Ann. Rheum Dis. 82, 1191–1204. doi: 10.1136/ard-2023-223859, PMID: 37328193

[B7] Chuah C. Jones M. K. Burke M. L. McManus D. P. Gobert G. N. (2014). Cellular and chemokine-mediated regulation in schistosome-induced hepatic pathology. Trends Parasitol. 30, 141–150. doi: 10.1016/j.pt.2013.12.009, PMID: 24433721

[B8] Diao H. Cheng N. Zhao Y. Xu H. Dong H. Thamm D. H.. (2019). Ivermectin inhibits canine mammary tumor growth by regulating cell cycle progression and WNT signaling. BMC Vet. Res. 15, 276. doi: 10.1186/s12917-019-2026-2, PMID: 31375107 PMC6679554

[B9] Ding H. Yang X. Tian J. Wang X. Ji Y. El-Ashram S.. (2021). JQ-1 ameliorates schistosomiasis liver fibrosis by suppressing JAK2 and STAT3 activation. BioMed. Pharmacother. 144, 112281. doi: 10.1016/j.biopha.2021.112281, PMID: 34624676

[B10] DiNicolantonio J. J. Barroso J. McCarty M. (2020). Ivermectin may be a clinically useful anti-inflammatory agent for late-stage COVID-19. Open Heart 7, e001350. doi: 10.1136/openhrt-2020-001350, PMID: 32895293 PMC7476419

[B11] Du K. Hyun J. Premont R. T. Choi S. S. Michelotti G. A. Swiderska-Syn M.. (2018). Hedgehog-YAP signaling pathway regulates glutaminolysis to control activation of hepatic stellate cells. Gastroenterology 154, 1465–1479.e13. doi: 10.1053/j.gastro.2017.12.022, PMID: 29305935 PMC5880682

[B12] Du K. Maeso-Díaz R. Oh S. H. Wang E. Chen T. Pan C.. (2023). Targeting YAP-mediated HSC death susceptibility and senescence for treatment of liver fibrosis. Hepatology 77, 1998–2015. doi: 10.1097/hep.0000000000000326, PMID: 36815382 PMC10416614

[B13] Ezhilarasan D. (2021). Mitochondria: A critical hub for hepatic stellate cells activation during chronic liver diseases. Hepatobiliary Pancreat Dis. Int. 20, 315–322. doi: 10.1016/j.hbpd.2021.04.010, PMID: 33975780

[B14] Garoffolo G. Casaburo M. Amadeo F. Salvi M. Bernava G. Piacentini L.. (2022). Reduction of cardiac fibrosis by interference with YAP-dependent transactivation. Circ. Res. 131, 239–257. doi: 10.1161/circresaha.121.319373, PMID: 35770662

[B15] Gordon S. Martinez F. O. (2010). Alternative activation of macrophages: mechanism and functions. Immunity 32, 593–604. doi: 10.1016/j.immuni.2010.05.007, PMID: 20510870

[B16] Gupta G. Khadem F. Uzonna J. E. (2019). Role of hepatic stellate cell (HSC)-derived cytokines in hepatic inflammation and immunity. Cytokine 124, 154542. doi: 10.1016/j.cyto.2018.09.004, PMID: 30241896

[B17] Haak A. J. Kostallari E. Sicard D. Ligresti G. Choi K. M. Caporarello N.. (2019). Selective YAP/TAZ inhibition in fibroblasts *via* dopamine receptor D1 agonism reverses fibrosis. Sci. Transl. Med. 11, eaau6296. doi: 10.1126/scitranslmed.aau6296, PMID: 31666402 PMC7066514

[B18] Hesse M. Modolell M. La Flamme A. C. Schito M. Fuentes J. M. Cheever A. W.. (2001). Differential regulation of nitric oxide synthase-2 and arginase-1 by type 1/type 2 cytokines *in vivo*: granulomatous pathology is shaped by the pattern of L-arginine metabolism. J. Immunol. 167, 6533–6544. doi: 10.4049/jimmunol.167.11.6533, PMID: 11714822

[B19] Huang Y. J. Yang C. K. Wei P. L. Huynh T. T. Whang-Peng J. Meng T. C.. (2017). Ovatodiolide suppresses colon tumorigenesis and prevents polarization of M2 tumor-associated macrophages through YAP oncogenic pathways. J. Hematol. Oncol. 10, 60. doi: 10.1186/s13045-017-0421-3, PMID: 28241877 PMC5329923

[B20] Jans D. A. Wagstaff K. M. (2020). Ivermectin as a broad-spectrum host-directed antiviral: the real deal? Cells 9, 2100. doi: 10.3390/cells9092100, PMID: 32942671 PMC7564151

[B21] Ju C. Pohl L. R. (2005). Tolerogenic role of Kupffer cells in immune-mediated adverse drug reactions. Toxicology 209, 109–112. doi: 10.1016/j.tox.2004.12.017, PMID: 15767021

[B22] Kamm D. R. McCommis K. S. (2022). Hepatic stellate cells in physiology and pathology. J. Physiol. 600, 1825–1837. doi: 10.1113/jp281061, PMID: 35307840 PMC9012702

[B23] Kisseleva T. Brenner D. (2021). Molecular and cellular mechanisms of liver fibrosis and its regression. Nat. Rev. Gastroenterol. Hepatol. 18, 151–166. doi: 10.1038/s41575-020-00372-7, PMID: 33128017

[B24] Laing R. Gillan V. Devaney E. (2017). Ivermectin - old drug, new tricks? Trends Parasitol. 33, 463–472. doi: 10.1016/j.pt.2017.02.004, PMID: 28285851 PMC5446326

[B25] Lange A. W. Sridharan A. Xu Y. Stripp B. R. Perl A. K. Whitsett J. A. (2015). Hippo/Yap signaling controls epithelial progenitor cell proliferation and differentiation in the embryonic and adult lung. J. Mol. Cell Biol. 7, 35–47. doi: 10.1093/jmcb/mju046, PMID: 25480985 PMC4400400

[B26] Lee D. E. Kang H. W. Kim S. Y. Kim M. J. Jeong J. W. Hong W. C.. (2022). Ivermectin and gemcitabine combination treatment induces apoptosis of pancreatic cancer cells *via* mitochondrial dysfunction. Front. Pharmacol. 13. doi: 10.3389/fphar.2022.934746, PMID: 36091811 PMC9459089

[B27] Li L. Zhou J. Li Q. Xu J. Qi J. Bian H. (2018). The inhibition of Hippo/Yap signaling pathway is required for magnesium isoglycyrrhizinate to ameliorate hepatic stellate cell inflammation and activation. BioMed. Pharmacother. 106, 83–91. doi: 10.1016/j.biopha.2018.06.102, PMID: 29957470

[B28] Liu Y. Meyer C. Müller A. Herweck F. Li Q. Müllenbach R.. (2011). IL-13 induces connective tissue growth factor in rat hepatic stellate cells *via* TGF-β-independent Smad signaling. J. Immunol. 187, 2814–2823. doi: 10.4049/jimmunol.1003260, PMID: 21804025

[B29] Liu P. Wang M. Lu X. D. Zhang S. J. Tang W. X. (2013). Schistosoma japonicum egg antigen up-regulates fibrogenesis and inhibits proliferation in primary hepatic stellate cells in a concentration-dependent manner. World J. Gastroenterol. 19, 1230–1238. doi: 10.3748/wjg.v19.i8.1230, PMID: 23482848 PMC3587479

[B30] Liu Z. Zhang L. Liang Y. Lu L. (2022). Pathology and molecular mechanisms of Schistosoma japonicum-associated liver fibrosis. Front. Cell Infect. Microbiol. 12. doi: 10.3389/fcimb.2022.1035765, PMID: 36389166 PMC9650140

[B31] Mannaerts I. Leite S. B. Verhulst S. Claerhout S. Eysackers N. Thoen L. F.. (2015). The Hippo pathway effector YAP controls mouse hepatic stellate cell activation. J. Hepatol. 63, 679–688. doi: 10.1016/j.jhep.2015.04.011, PMID: 25908270

[B32] McManus D. P. Bergquist R. Cai P. Ranasinghe S. Tebeje B. M. You H. (2020). Schistosomiasis-from immunopathology to vaccines. Semin. Immunopathol. 42, 355–371. doi: 10.1007/s00281-020-00789-x, PMID: 32076812 PMC7223304

[B33] Meyersburg D. Welponer T. Kaiser A. Selhofer S. Tatarski R. Handisurya A.. (2023). Comparison of topical benzyl benzoate vs. oral ivermectin in treating scabies: A randomized study. J. Eur. Acad. Dermatol. Venereol 37, 160–165. doi: 10.1111/jdv.18573, PMID: 36097258 PMC10087012

[B34] Moroishi T. Hansen C. G. Guan K. L. (2015). The emerging roles of YAP and TAZ in cancer. Nat. Rev. Cancer 15, 73–79. doi: 10.1038/nrc3876, PMID: 25592648 PMC4562315

[B35] Müller H. Straßmann J. K. Baier A. S. von Bülow V. Stettler F. Hagen M. J.. (2024). Liver fibrosis is enhanced by a higher egg burden in younger mice infected with S. mansoni. Cells 13, 1643. doi: 10.3390/cells13191643, PMID: 39404406 PMC11475498

[B36] Nishio M. Sugimachi K. Goto H. Wang J. Morikawa T. Miyachi Y.. (2016). Dysregulated YAP1/TAZ and TGF-β signaling mediate hepatocarcinogenesis in Mob1a/1b-deficient mice. Proc. Natl. Acad. Sci. U.S.A. 113, E71–E80. doi: 10.1073/pnas.1517188113, PMID: 26699479 PMC4711826

[B37] Piccolo S. Dupont S. Cordenonsi M. (2014). The biology of YAP/TAZ: hippo signaling and beyond. Physiol. Rev. 94, 1287–1312. doi: 10.1152/physrev.00005.2014, PMID: 25287865

[B38] Sandler N. G. Mentink-Kane M. M. Cheever A. W. Wynn T. A. (2003). Global gene expression profiles during acute pathogen-induced pulmonary inflammation reveal divergent roles for Th1 and Th2 responses in tissue repair. J. Immunol. 171, 3655–3667. doi: 10.4049/jimmunol.171.7.3655, PMID: 14500663

[B39] She G. Du J. C. Wu W. Pu T. T. Zhang Y. Bai R. Y.. (2023). Hippo pathway activation mediates chemotherapy-induced anti-cancer effect and cardiomyopathy through causing mitochondrial damage and dysfunction. Theranostics 13, 560–577. doi: 10.7150/thno.79227, PMID: 36632235 PMC9830444

[B40] Sunami Y. Leithäuser F. Gul S. Fiedler K. Güldiken N. Espenlaub S.. (2012). Hepatic activation of IKK/NFκB signaling induces liver fibrosis *via* macrophage-mediated chronic inflammation. Hepatology 56, 1117–1128. doi: 10.1002/hep.25711, PMID: 22407857

[B41] Tacke F. (2017). Targeting hepatic macrophages to treat liver diseases. J. Hepatol. 66, 1300–1312. doi: 10.1016/j.jhep.2017.02.026, PMID: 28267621

[B42] von Bülow V. Schneider M. Dreizler D. Russ L. Baier A. Buss N.. (2024). Schistosoma mansoni-induced oxidative stress triggers hepatocellular proliferation. Cell Mol. Gastroenterol. Hepatol. 17, 107–117. doi: 10.1016/j.jcmgh.2023.08.014, PMID: 37696392 PMC10665951

[B43] Wen Z. Ji X. Tang J. Lin G. Xiao L. Liang C.. (2017). Positive feedback regulation between transglutaminase 2 and toll-like receptor 4 signaling in hepatic stellate cells correlates with liver fibrosis post schistosoma japonicum infection. Front. Immunol. 8. doi: 10.3389/fimmu.2017.01808, PMID: 29321784 PMC5733538

[B44] Xiang D. Zou J. Zhu X. Chen X. Luo J. Kong L.. (2020). Physalin D attenuates hepatic stellate cell activation and liver fibrosis by blocking TGF-β/Smad and YAP signaling. Phytomedicine 78, 153294. doi: 10.1016/j.phymed.2020.153294, PMID: 32771890

[B45] Xu F. Liu C. Zhou D. Zhang L. (2016). TGF-β/SMAD pathway and its regulation in hepatic fibrosis. J. Histochem Cytochem 64, 157–167. doi: 10.1369/0022155415627681, PMID: 26747705 PMC4810800

[B46] Yang S. N. Y. Atkinson S. C. Wang C. Lee A. Bogoyevitch M. A. Borg N. A.. (2020). The broad spectrum antiviral ivermectin targets the host nuclear transport importin α/β1 heterodimer. Antiviral Res. 177, 104760. doi: 10.1016/j.antiviral.2020.104760, PMID: 32135219

[B47] Yimlamai D. Christodoulou C. Galli G. G. Yanger K. Pepe-Mooney B. Gurung B.. (2014). Hippo pathway activity influences liver cell fate. Cell 157, 1324–1338. doi: 10.1016/j.cell.2014.03.060, PMID: 24906150 PMC4136468

[B48] You H. Wang X. Ma L. Zhang F. Zhang H. Wang Y.. (2023). Insights into the impact of hepatitis B virus on hepatic stellate cell activation. Cell Commun. Signal 21, 70. doi: 10.1186/s12964-023-01091-7, PMID: 37041599 PMC10088164

[B49] Zanconato F. Cordenonsi M. Piccolo S. (2016). YAP/TAZ at the roots of cancer. Cancer Cell 29, 783–803. doi: 10.1016/j.ccell.2016.05.005, PMID: 27300434 PMC6186419

[B50] Zhang T. He X. Caldwell L. Goru S. K. Ulloa Severino L. Tolosa M. F.. (2022). NUAK1 promotes organ fibrosis *via* YAP and TGF-β/SMAD signaling. Sci. Transl. Med. 14, eaaz4028. doi: 10.1126/scitranslmed.aaz4028, PMID: 35320001

[B51] Zhu T. Ma Z. Wang H. Jia X. Wu Y. Fu L.. (2020). YAP/TAZ affects the development of pulmonary fibrosis by regulating multiple signaling pathways. Mol. Cell Biochem. 475, 137–149. doi: 10.1007/s11010-020-03866-9, PMID: 32813142

